# TRAF6 Promotes PRMT5 Activity in a Ubiquitination-Dependent Manner

**DOI:** 10.3390/cancers15092501

**Published:** 2023-04-27

**Authors:** Liu Liu, Shasha Yin, Wenjian Gan

**Affiliations:** Department of Biochemistry and Molecular Biology, Medical University of South Carolina, Charleston, SC 29425, USA

**Keywords:** PRMT5, TRAF6, ubiquitination, arginine methylation, cell proliferation, cancer

## Abstract

**Simple Summary:**

PRMT5 is overexpressed and activated in various human cancers, including breast cancer. This study aims to dissect the mechanism underlying how PRMT5 is dysregulated in cancers. Our results demonstrate that TRAF6-mediated ubiquitination plays an important role in the regulation of PRMT5 activity and cell proliferation. Thus, inhibition of TRAF6 is a possible strategy for improving PRMT5 targeted therapy.

**Abstract:**

Protein arginine methyltransferase 5 (PRMT5) is the primary enzyme generating symmetric dimethylarginine (sDMA) on numerous substrates, through which it regulates many cellular processes, such as transcription and DNA repair. Aberrant expression and activation of PRMT5 is frequently observed in various human cancers and associated with poor prognosis and survival. However, the regulatory mechanisms of PRMT5 remain poorly understood. Here, we report that TRAF6 serves as an upstream E3 ubiquitin ligase to promote PRMT5 ubiquitination and activation. We find that TRAF6 catalyzes K63-linked ubiquitination of PRMT5 and interacts with PRMT5 in a TRAF6-binding-motif-dependent manner. Moreover, we identify six lysine residues located at the N-terminus as the primarily ubiquitinated sites. Disruption of TRAF6-mediated ubiquitination decreases PRMT5 methyltransferase activity towards H4R3 in part by impairing PRMT5 interaction with its co-factor MEP50. As a result, mutating the TRAF6-binding motifs or the six lysine residues significantly suppresses cell proliferation and tumor growth. Lastly, we show that TRAF6 inhibitor enhances cellular sensitivity to PRMT5 inhibitor. Therefore, our study reveals a critical regulatory mechanism of PRMT5 in cancers.

## 1. Introduction

Arginine methylation is acknowledged as one of the most common post-translational modifications (PTMs). It occurs on approximately 7% of all arginine residues in arginine-methylated proteins, which is comparable to phosphorylation at 9% of serine residues and ubiquitination at 7% of lysine residues [1,2,3,4]. Extensive studies have demonstrated that protein arginine methylation plays an important role in a variety of fundamental cellular processes including DNA replication [5], transcription [6], RNA processing [7], DNA repair [8], and protein degradation [9].

Protein arginine methyltransferases (PRMTs) are the writers responsible for transferring the methyl group to the guanidino nitrogen atoms of arginine, forming three types of methylated arginine residue: monomethylarginine (MMA), asymmetric dimethylarginine (aDMA), and symmetric dimethylarginine (sDMA) [10,11]. In mammalian cells, nine members of the PRMT family have been identified, all of which share a highly homologous SAM-dependent methyltransferase domain. According to their catalytic activity, these PRMTs can be classified into three distinct types: type I contains six members (PRMT1, 2, 3, 4, 6, and 8), type II includes two members (PRMT5 and PRMT9), and type III has only one member (PRMT7). Type I/II PRMTs can catalyze the formation of MMA intermediate, and then further catalyze the formation of aDMA and sDMA, respectively, while the type III enzyme only catalyzes the formation of MMA [12].

PRMT5 is the main type II enzyme that deposits the sDMA on histones and non-histone substrates [13]. PRMT5 was first characterized as a transcriptional repressor by catalyzing the symmetric dimethylation of histone H3 arginine 8 (H3R8) and H4 arginine 3 (H4R3), leading to transcriptional repression of tumor-suppressor genes [14]. PRMT5 also regulates the transcriptional program by methylating transcription factors, mRNA splicing factors, and RNA binding proteins [15]. Moreover, PRMT5 regulates several oncogenic signaling pathways, including RAS-ERK, NF-κB, and AKT pathways [16,17,18]. Compared to the extensive studies on its downstream substrates, the upstream regulators of PRMT5 remain largely unknown.

Ubiquitination is another of the most common PTMs which covalently conjugates the small protein ubiquitin (Ub) to the lysine residue through a sequential enzymatic cascade consisting of ubiquitin-activating enzymes (E1s), ubiquitin-conjugating enzymes (E2s), and E3 ubiquitin ligases (E3s) [19,20]. Ubiquitin can form seven types of ubiquitin linkages on substrates through seven lysine residues (K6, K11, K27, K29, K33, K48, and K63) and serves as different signals to control protein functions. It is widely accepted that K11- and K48-ubiquitin linkages are proteasomal degradation markers, while K63-ubiquitin linkage serves as a non-proteolytic signal to regulate enzymatic activity, protein cellular localization, and protein–protein interactions [21,22].

Here, we investigated the interplay between PRMT5 and ubiquitination. Our findings demonstrate that TRAF6 catalyzes non-proteolytic K63-linked ubiquitination of PRMT5 and consequently promotes PRMT5 activity and its role in cell proliferation and tumorigenesis.

## 2. Materials and Methods

### 2.1. Cell Culture and Reagents

HEK293T, MCF-7, and MDA-MB-231 cells were obtained from American Type Culture Collection. All cell lines were maintained in Dulbecco’s Modified Eagle’s Medium (DMEM) containing 10% fetal bovine serum (FBS), 100 units/mL penicillin, and 100 μg/mL streptomycin. GSK3326595 (HY-101563) and C25-140 (HY-120934) were purchased from MedChemExpress (Monmouth Junction, NJ, USA).

### 2.2. Transfection and Viral Infection

For protein expression in cells, transfection was performed using Lipofectamine 3000 and Plus reagents following manufacturer’s instructions. For lentiviral virus production, 293T cells were transfected with target and packaging plasmids (pMD2G and psPAX2) with Polyethylenimine (PEI). Twenty-four hours post-transfection, fresh medium was replaced. Virus-containing supernatants were harvested at forty-eight hours post-transfection and filtered with 0.45 μm PES filter. Targeted cells were infected with viruses and selected with hygromycin (200 μg/mL), puromycin (2 μg/mL), or blasticidin (10 μg/mL) for 4 days to eliminate the non-infected cells.

### 2.3. Plasmids

HA-TRAF6, HA-PRMT1, HA-PRMT5, HA-PRMT5-ΔC, and HA-PRMT5-ΔN were generated by cloning the corresponding cDNA into the pRK5-HA vector. HA-TRAF1, HA-TRAF2, and HA-TRAF3 were generated by cloning the corresponding cDNA into the pCDNA3-HA vector. Myc-TRAF2 and Myc-TRAF6 were generated by cloning the corresponding cDNA into the pCDNA3-Myc vector. Flag-TRAF6 and Flag-PRMT5 were generated by cloning the corresponding cDNA into the pRK5-Flag vector. Flag-Itch, Flag-WWP1, and Flag-WWP2 were generated by cloning the corresponding cDNA into the pFlag-CMV2 vector. Inducible TRAF6-expressing constructs were generated by cloning the corresponding cDNA into the pTRIPZ vector. Various His-Ub constructs were generated as previously described [23,24]. Various PRMT5 mutations were generated using the QuikChange XL site-directed mutagenesis kit (20518, Santa Clara, CA, USA). Single-guide RNAs (sgRNAs) targeting TRAF6 and PRMT5 were designed at https://www.synthego.com (accessed on 13 March 2023) and were cloned into the lentiCRISPR v2 vector (Addgene, 52961). PRMT5 sgRNA was described previously [18]. TRAF6-sg1, 5′-GTAACAAAAGATGATAGTGT-3′; TRAF6-sg2, 5′-ACATTCTGAAGGAT TGTCCA-3′.

### 2.4. Antibodies

All primary antibodies were used at a dilution of 1:1000–3000 in TBST buffer with 5% non-fat milk for Western blotting. Anti-PRMT5 (79998), anti-TRAF6 (8028S), anti-MEP50 (2018S), rabbit anti-Myc-tag (2278), and rabbit anti-HA (3724) were purchased from Cell Signaling Technology (Danvers, MA, USA). Mouse monoclonal antibody to HA (901503) was purchased from Biolegend (San Diego, CA, USA). Rabbit anti-Flag (F7425), mouse anti-Flag (F3165), peroxidase-conjugated anti-mouse secondary antibody (A4416), and anti-rabbit secondary antibody (A4914) were purchased from Sigma Aldrich (St. Louis, MO, USA). Anti-H4R3me2s (A-3718-100) was purchased from EpigenTek (Farmingdale, NY, USA). Anti-Tubulin (66031-1-Ig) and anti-H4 (16047-1-AP) were purchased from Proteintech (Rosemont, IL, USA).

### 2.5. Immunoblot (IB) and Immunoprecipitation (IP) Analyses

Cells were washed with cold phosphate-buffered saline (PBS) and lysed in EBC buffer (50 mM Tris pH 7.5, 120 mM NaCl and 0.5% NP-40) supplemented with protease inhibitors (K1019, APExBIO, Houston, TX, USA) and phosphatase inhibitors (K1015, APExBIO, Houston, USA). The cell lysates were clarified by centrifugation at 13,200 r.p.m. at 4 °C for 10 min. The protein concentrations of lysates were measured using Nanodrop with Bio-Rad protein assay reagent. Equal amounts of whole-cell lysates were resolved by SDS-PAGE and immunoblotted with indicated antibodies. For immunoprecipitation, 2000–5000 μg lysates were incubated with agarose-conjugated primary antibodies for 3–4 h at 4 °C or with the indicated primary antibody (3 to 5 μg) overnight followed by 1 h incubation with Protein A Sepharose beads. Immunoprecipitants were washed three times with NETN buffer (20 mM Tris, pH 8.0, 150 mM NaCl, 1 mM EDTA, and 0.5% NP-40) and then resolved by SDS-PAGE. Anti-HA agarose beads (A2095) and anti-Flag agarose beads (A2220) were purchased from Sigma Aldrich. Anti-Myc agarose beads (658502) were purchased from BioLegend. Anti-PRMT5 antibody conjugated to agarose (sc-376937) was purchased from Santa Cruz Biotechnology (Dallas, TX, USA).

### 2.6. In Vivo Ubiquitination Assays

In vivo ubiquitination assay was performed as previously described [25]. Cells were transfected with His-Ub, PRMT5, and E3 ubiquitin ligase constructs. Forty-eight hours post-transfection, cells were directly lysed in buffer A (6 M guanidine-HCl, 0.1 M Na_2_HPO_4_/NaH_2_PO_4_, and 10 mM imidazole [pH 8.0]) and sonicated. The lysates were incubated with nickel–nitrilotriacetic acid (Ni-NTA) matrices (QIAGEN, Germantown, MD, USA) for three hours at room temperature. The His pull-down products were washed twice with buffer A, twice with buffer A/TI (1 volume buffer A and 3 volumes buffer TI), and one time with buffer TI (25 mM Tris-HCl and 20 mM imidazole [pH 6.8]). The pull-down proteins were resolved by SDS-PAGE for immunoblotting.

### 2.7. In Vitro Histone Arginine Methylation Assays

In vitro methylation assays were performed as previously described [18]. Briefly, 1 μg of recombinant Histone H4 (M2504S, NEB, Ipswich, MA, USA) was incubated with PRMT5 immunoprecipitated from cells in the methylation buffer (50 mM Tris pH 8.5, 20 mM KCl, 10 mM MgCl_2_, 1 mM β-mercaptoethanol, and 100 mM sucrose) with 1 μL S-adenosylmethionine (B9003S, NEB) at 30 °C for 1 h, stopped by adding 3× SDS loading buffer, resolved by SDS-PAGE, and immunoblotted with H4R3me2s antibody.

### 2.8. Cell Viability Assays

Cells were seeded in 96-well plates (1000–2000 cells/well) for 24 h and treated with TRAF6 inhibitor and/or PRMT5 inhibitor for 96 h. Assays were performed using the Cell Titer-Glo luminescent cell viability assay kit according to the manufacturer’s instructions (G7572, Promega, Madison, WI, USA).

### 2.9. Colony Formation Assays

Cells were seeded in 6-well plates (200–300 cells/well) and cultured for 10 to 15 days until formation of visible colonies. Colonies were washed with PBS and fixed with 10% acetic acid/10% methanol for 20 min, then stained with 0.4% crystal violet for 20 min. After staining, the plates were washed with distilled water and air-dried. The colonies were counted manually.

### 2.10. Mouse Xenograft Assays

MDA-MB-231 cells depleted of endogenous PRMT5 and then stably expressing PRMT5-WT or PRMT5-3A mutants were injected into the flank of 5-week-old female nude mice (007850, The Jackson Laboratory, Bar Harbor, USA). Tumor size was measured every other day with an electronic caliper. The tumor volume was calculated by *L* × *W*^2^ × 0.5, where L is the longest diameter and W is the shortest diameter. Mice were euthanized at day 23 and the solid tumors were dissected. All mice are housed in 22 °C, 50–60% humidity, and a 12 h light/12 h dark cycle. All mouse experiments were conducted under Protocol IACUC-2018-00604-1 approved by the MUSC Institutional Animal Care and Use Committee.

### 2.11. Statistical Analysis

All quantitative data are presented as the means ± SD of three biologically independent experiments. Statistical analyses were performed using GraphPad Prism 9 (GraphPad, Boston, USA). Significance was determined by two-tailed Student’s *t* test or two-way analysis of variance (ANOVA). *p* < 0.05 was considered significant.

## 3. Results

### 3.1. TRAF6 Promotes K63-Linked Ubiquitination of PRMT5

**Figure 1:**
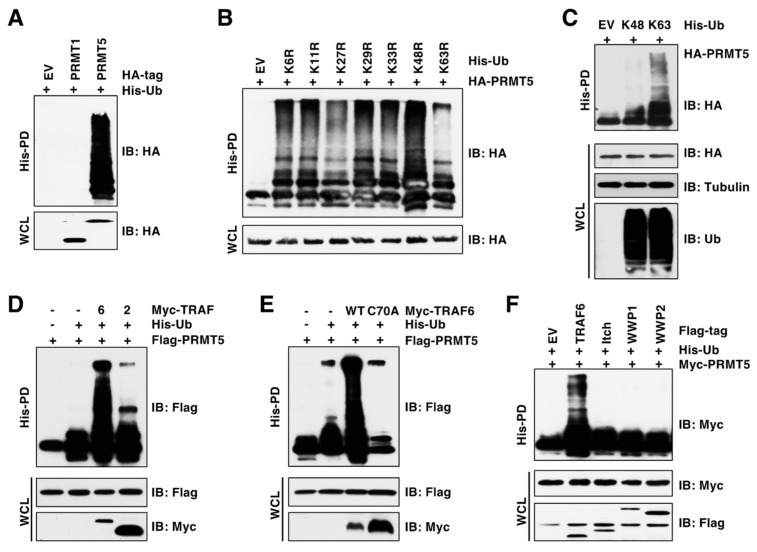
TRAF6 promotes K63-linked ubiquitination of PRMT5. (**A**) In vivo ubiquitination assay examining PRMT1 and PRMT5 ubiquitination. Immunoblot (IB) analysis of whole-cell lysate (WCL) and His pull-down (His-PD) products derived from HEK293T cells transfected with indicated plasmids. (**B**,**C**) IB analysis of WCL and His-PD products derived from HEK293T cells transfected with HA-PRMT5 and His-tagged ubiquitin mutants. (**D**) IB analysis of WCL and His-PD products derived from HEK293T cells transfected with Flag-PRMT5, His-Ub, and Myc-TRAF2 or TRAF6 plasmids. (**E**) IB analysis of WCL and His-PD products derived from HEK293T cells transfected with Flag-PRMT5, His-Ub, and Myc-TRAF6-WT or C70A plasmids. (**F**) IB analysis of WCL and His-PD products derived from HEK293T cells transfected with indicated plasmids. The original blots image for Figure 1 is available in Supplementary Materials.

Ubiquitination is one of the most common PTMs and serves as a key mechanism controlling protein functions [26]. However, whether and how PRMT5 is regulated by ubiquitination remains largely unknown. To address this question, we performed in vivo ubiquitination assay under denaturing conditions, which disrupts PRMT5 interactions with all its binding partners to exclude possible interferences from their ubiquitination. Strikingly, compared to PRMT1 (the main type I enzyme), PRMT5 was heavily ubiquitinated in cells (Figure 1A). To identify the ubiquitin linkage of PRMT5, we co-transfected PRMT5 with a panel of ubiquitin mutants, in which the indicated lysine (K) was mutated to arginine (R) [24]. The ubiquitin K63R mutation, and to a lesser extent the K27R mutation, decreased PRMT5 ubiquitination (Figure 1B). In contrast, co-transfection of PRMT5 with K63-only ubiquitin—but not K48-only ubiquitin, in which only the indicated lysine is not mutated—conferred PRMT5 ubiquitination (Figure 1C). To identify the E3 ubiquitin ligase(s) responsible for the K63-linked ubiquitination of PRMT5, we focused on the E3 ligases of TRAF and NEDD4 families, which prefer to catalyze ubiquitination with the K63 linkage [27,28]. We found that TRAF6, but not its close member TRAF2 or the E3-ligase-inactive mutant TRAF6-C70A [29], promoted PRMT5 ubiquitination (Figure 1D,E). Compared to TRAF6, the members of the NEDD4 family, including Itch, WWP1, and WWP2, failed to promote PRMT5 ubiquitination (Figure 1F). These results suggest that TRAF6 is the main E3 ubiquitin ligase responsible for K63-linked ubiquitination of PRMT5.

### 3.2. TRAF6 Specifically Binds to PRMT5 through a Conserved Binding Motif

**Figure 2:**
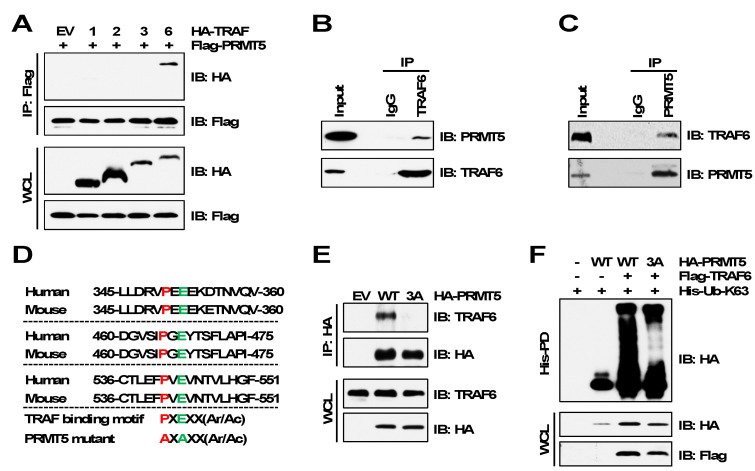
TRAF6 interacts with PRMT5 via the TRAF6-binding motifs. (**A**) IB analysis of WCL and Flag immunoprecipitation (IP) products derived from HEK293T cells transfected with Flag-PRMT5 and HA-tagged TRAF plasmids. (**B**,**C**) IB analysis of Input and TRAF6 IP or PRMT5 IP products derived from MDA-MB-231 cells. (**D**) Protein sequence of TRAF6-binding motifs of PRMT5. (**E**) IB analysis of WCL and HA IP products derived from MDA-MB-231 cells stably expressing HA-PRMT5-WT or HA-PRMT5-3A. EV (empty vector) is a negative control. (**F**) IB analysis of WCL and His-PD products derived from HEK293T cells transfected with indicated plasmids. The original blots image for Figure 2 is available in Supplementary Materials.

To further validate TRAF6 is the E3 ubiquitin ligase of PRMT5, we examined their interactions by immunoprecipitation assays. In agreement with TRAF6 promoting PRMT5 ubiquitination, TRAF6, but not other members of the TRAF family, interacted with PRMT5 (Figure 2A). We further confirmed the endogenous interaction between TRAF6 and PRMT5 (Figure 2B,C). Structural studies have revealed that TRAF6 binds to its substrates through a motif containing six amino acids, PxExxZ (P: proline, E: glutamic acid, x: any amino acid, Z: acidic or aromatic amino acid) [30,31]. Following a closer examination of the PRMT5 protein sequence, we identified three putative TRAF6-binding motifs (Figure 2D), mutation of which (PxE to AxA, called PRMT5-3A) abolished the PRMT5-binding of TRAF6 (Figure 2E). As a result, TRAF6-mediated ubiquitination of PRMT5-3A was severely decreased compared to PRMT5-WT (Figure 2F). These results suggest that the TRAF6-binding motifs are required for TRAF6 interaction with PRMT5.

### 3.3. TRAF6 Promotes Ubiquitination of PRMT5 on Multiple Lysine Residues

**Figure 3:**
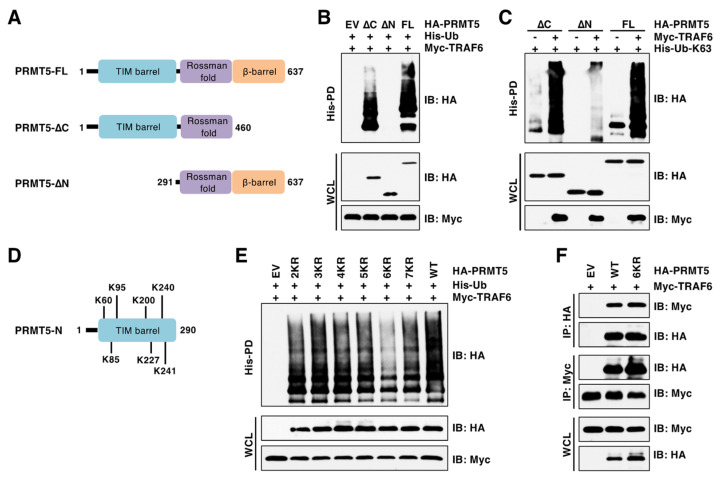
TRAF6 ubiquitinates six lysine residues at the N-terminus of PRMT5. (**A**) A schematic presentation of PRMT5 domains and truncated PRMT5. (**B**,**C**) IB analysis of WCL and His-PD products derived from HEK293T cells transfected with indicated plasmids. (**D**) The putative ubiquitinated lysine (K) residues in the N-terminus of PRMT5. (**E**) IB analysis of WCL and His-PD products derived from HEK293T cells transfected with indicated plasmids. (**F**) IB analysis of WCL and HA/Myc IP products derived from HEK293T cells transfected with indicated plasmids. The original blots image for Figure 3 is available in Supplementary Materials.

Integrated proteomic analysis by mass spectrometry identified at least 13 lysine (K) residues of PRMT5 being ubiquitinated [3]. However, the E3 ubiquitin ligases responsible for PRMT5 ubiquitination at these sites have not yet been identified. To identify putative ubiquitinated sites by TRAF6, we generated various truncated forms of PRMT5 according to its structural domains (Figure 3A) [32]. Deletion of the N-terminal TIM barrel domain (1–290 aa), but not deletion of C-terminus β-barrel domain (461–637 aa), abrogated TRAF6-mediated K63-linked ubiquitination of PRMT5 (Figure 3B,C). The TIM barrel domain contains seven ubiquitinated K residues (Figure 3D). We generated various lysine-to-arginine (KR) mutations. Of these, the 6KR mutant (K85/K95/K200/K227/K240/K241) largely abolished PRMT5 ubiquitination by TRAF6 (Figure 3E), although this PRMT5 6KR mutant was capable of interaction with TRAF6 at a level comparable to PRMT5-WT (Figure 3F). Interestingly, the 7KR mutant displayed higher ubiquitination signals than the 6KR mutant (Figure 3E). It is possible that the 7KR mutant might cause PRMT5 conformational changes and consequently enhance its binding to other E3 ubiquitin ligases, which will be further studied in the future. These data demonstrated that PRMT5 is ubiquitinated at multiple lysine residues by TRAF6.

### 3.4. TRAF6-Mediated PRMT5 Ubiquitination Promotes Its Activity

**Figure 4:**
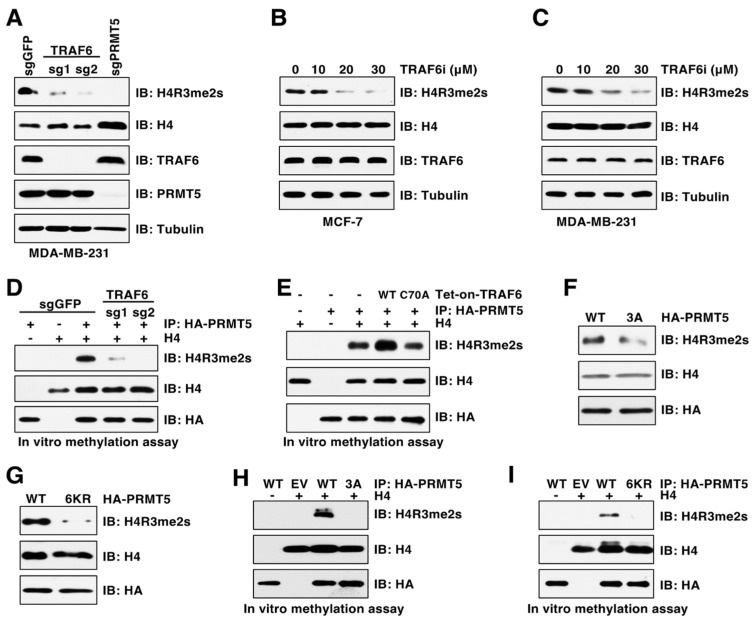
PRMT5 ubiquitination is critical for its activity. (**A**) IB analysis of WCL and histones derived from MDA-MB-231 cells infected with viruses expressing control sgGFP, TRAF6 sgRNA, or PRMT5 sgRNA. (**B**,**C**) IB analysis of WCL and histones derived from cells treated with indicated TRAF6 inhibitor, C25–140, for 3 days. (**D**,**E**) In vitro arginine methylation assays using recombinant histone H4 as substrate. (**F**,**G**) IB analysis of WCL and histones derived from MDA-MB-231 cells expressing HA-PRMT5-WT, 3A or 6KR. (**H**,**I**) In vitro arginine methylation assays using recombinant histone H4 as substrate. The original blots image for Figure 4 is available in Supplementary Materials.

Given that ubiquitination is a key mechanism regulating enzymatic activity [21,22], we were determined to explore whether TRAF6 regulates PRMT5 methyltransferase activity by examining symmetric dimethylation of histone H4R3 (H4R3me2s), which is a well-characterized PRMT5 substrate and a widely used marker of PRMT5 activation [14]. We found that depletion of TRAF6 by CSISPR-Cas9 severely decreased H4R3me2s levels in cells (Figure 4A). Consistently, the TRAF6 inhibitor C25–140, which inhibits TRAF6 E3 ubiquitin ligase activity by disrupting its binding to the E2 Ubc13 [33], decreased H4R3me2s in a dose-dependent manner in cells (Figure 4B,C). In agreement with these results, in vitro arginine methylation assay showed that PRMT5 purified from TRAF6-depleted cells displayed much lower methyltransferase activity towards H4R3 compared to PRMT5 purified from control cells (Figure 4D). In contrast, overexpression of TRAF6-WT, but not the C70A mutant, enhanced PRMT5 activity in methylating H4R3me2s in vitro (Figure 4E). Importantly, compared to cells expressing PRMT5-WT, cells expressing the PRMT5-3A or PRMT5-6KR mutants displayed a significant decrease in H4R3me2s (Figure 4F,G), which was further validated by the in vitro arginine methylation assays (Figure 4H,I). These results suggest that TRAF6-mediated ubiquitination is critical for PRMT5 activity.

### 3.5. Deficiency in Ubiquitination Impairs PRMT5 Binding of MEP50

**Figure 5:**
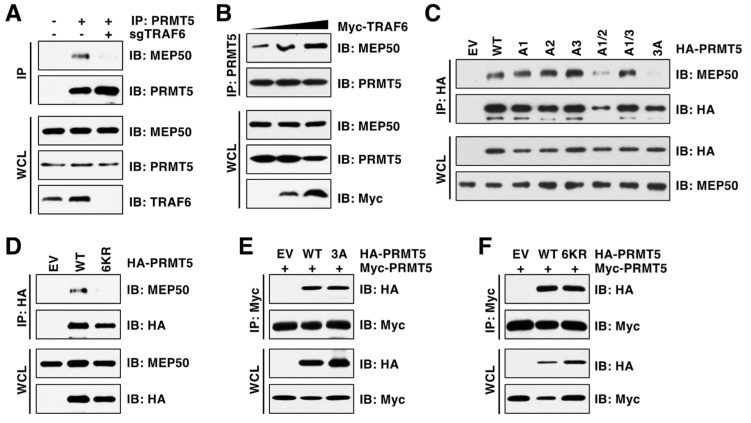
PRMT5 ubiquitination is required for its interaction with MEP50. (**A**) IB analysis of WCL and PRMT5 IP derived from MDA-MB-231 cells with or without TRAF6 depletion. (**B**) IB analysis of WCL and PRMT5 IP derived from HEK293T cells transfected with TRAF6. (**C**,**D**) IB analysis of WCL and HA IP derived from HEK293T cells transfected with indicated plasmids. (**E**,**F**) IB analysis of WCL and Myc IP derived from HEK293T cells transfected with indicated plasmids. The original blots image for Figure 5 is available in Supplementary Materials.

We then aimed to understand the molecular mechanism by which TRAF6-mediated ubiquitination triggers PRMT5 activation. Generally, K63-linked ubiquitination regulates protein interaction or cellular localization, controlling protein activity and substrate specificity in turn [34]. Structural studies revealed that PRMT5 and MEP50 (also called WDR77) form a core hetero-octameric complex through the N-terminal TIM barrel domain [32], where the ubiquitinated lysine residues are located (Figure 3D). We found that depletion of TRAF6 reduced PRMT5 interaction with MEP50 (Figure 5A). In contrast, ectopic expression of TRAF6 enhanced their interactions (Figure 5B). Notably, only the PRMT5-3A mutation, but not the individual or double mutations, impaired PRMT5-binding of MEP50 (Figure 5C). Moreover, the PRMT5-6KR mutant, which is deficient in ubiquitination, displayed much weaker interaction with MEP50 than PRMT5-WT (Figure 5D). Of note, neither PRMT5-3A nor PRMT5-6KR mutants affected PRMT5 dimer formation (Figure 5E,F), suggesting that these mutants did not cause a global conformation change. These results together demonstrate that TRAF6-mediated ubiquitination promotes PRMT5 activity in part by facilitating the PRMT5-MEP50 complex formation.

### 3.6. Ubiquitination of PRMT5 Is Critical for Breast Cancer Cell Proliferation

**Figure 6:**
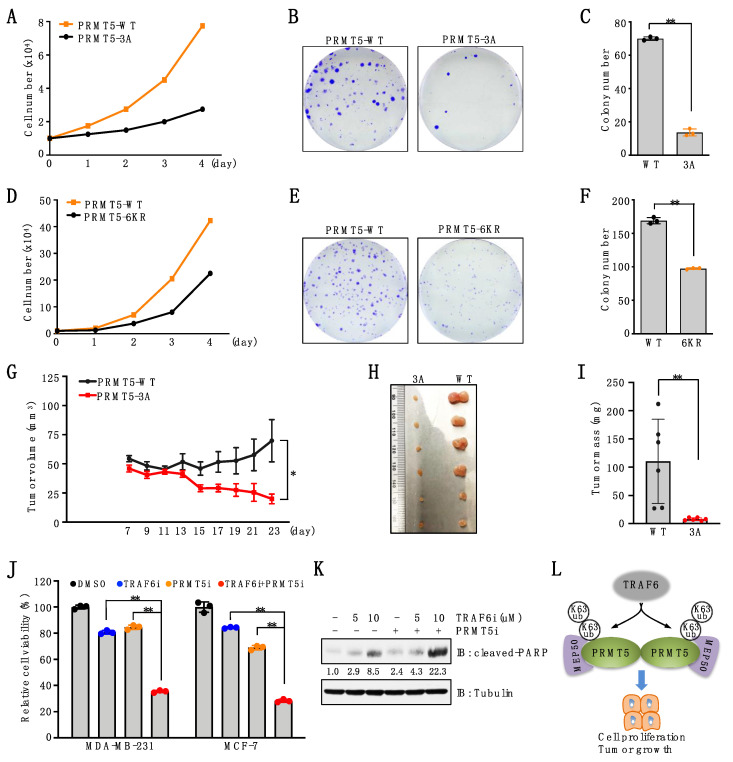
Deficiency in PRMT5 ubiquitination suppresses cell proliferation and tumor growth. (**A**) MDA-MB-231 cells stably expressing PRMT5-WT or PRMT5-3A were subjected to cell proliferation assays. (**B**,**C**) MDA-MB-231 cells stably expressing PRMT5-WT or PRMT5-3A were subjected to colony formation assays. Representative images are shown in (**B**). Colonies are quantified in (**C**). Data are means ± SD (*n* = 3). ** *p* < 0.01, Student’s *t*-test. (**D**) MDA-MB-231 cells stably expressing PRMT5-WT or PRMT5-6KR were subjected to cell proliferation assays. (**E**,**F**) MDA-MB-231 cells stably expressing PRMT5-WT or PRMT5-6KR were subjected to colony formation assays. Representative images were shown in (**E**). Colonies were quantified in (**F**). Data are means ± SD (n = 3). ** *p* < 0.01, Student’s *t*-test. (**G**–**I**) PRMT5-depleted MDA-MB-231 cells stably expressing PRMT5-WT or 3A mutants were subjected to mouse xenograft assays. Tumor growth was monitored (G) and dissected tumors were weighed (H, I). Data are shown as the mean ± SEM of *n* = 6 mice. (**J**) Cells were treated with 10 μM TRAF6 inhibitor (C25–140), 1 μM PRMT5 inhibitor (GSK3326595), or both for 4 days and then subjected to cell viability assays. Data are means ± SD (n = 3). ** *p* < 0.01, one-way ANOVA. (**K**) IB analysis of WCL derived from MDA-MB-231 cells treated with 1 μM PRMT5 inhibitor, 5 μM or 10 μM TRAF6 inhibitor for 3 days. (**L**) A proposed model depicting TRAF6-mediated K63-linked ubiquitination in regulating PRMT5 activation and cell proliferation. The original blots image for Figure 6 is available in Supplementary Materials.

Our previous study demonstrated that depletion of PRMT5 significantly suppressed breast cancer cell proliferation and tumor growth [18]. To investigate the biological function of TRAF6-mediated PRMT5 ubiquitination, we reintroduced PRMT5-WT, PRMT5-3A, or PRMT5-6KR into PRMT5-depleted breast cancer cells. Cell proliferation assays showed that, compared to cells expressing PRMT5-WT, cells expressing PRMT5-3A or PRMT5-6KR displayed a significant decrease in cell proliferation (Figure 6A,D). Consistently, colony formation was significantly decreased in cells expressing these PRMT5 mutants compared to PRMT5-WT (Figure 6B,C,E,F). Moreover, the mouse xenograft assay showed that compared to PRMT5-WT, PRMT5-3A mutation significantly suppressed tumor growth (Figure 6G–I). Lastly, we found that, compared to the single agent, co-treatment of TRAF6 and PRMT5 inhibitors significantly decreased cell viability in breast cancer cells (Figure 6J), which is in part achieved by enhancing cell apoptosis (Figure 6K). These data suggest that TRAF6-mediated ubiquitination of PRMT5 is important for its proliferative function in breast cancer cells.

## 4. Discussion

A growing amount of evidence reveals that PRMT5 is overexpressed or activated in a variety of cancers [35]. Whole-genome DNA sequencing revealed that genetic alterations including amplification, mutation, and deletion are rare in the *PRMT5* gene [36], suggesting that PRMT5 is mainly regulated at post-transcriptional and post-translational levels. Several microRNAs, including miR-19a, miR-25, miR-32, miR-92b, and miR-96, have been identified to bind to the 3′-untranslated region (3′UTR) of PRMT5 mRNA and inhibit PRMT5 translation in lymphoid cancer cells [14,37]. Notably, proteomic analysis reveals that PRMT5 undergoes multiple post-translational modifications, including phosphorylation, ubiquitination, methylation, and acetylation [3], indicating a critical role of PTMs in modulating PRMT5 function. In particular, multiple studies have documented the regulatory role of phosphorylation in modulating PRMT5 activity and substrate specificity. JAK2V617F-mediated phosphorylation of PRMT5 disrupted its interaction with MEP50, resulting in inhibition of its methyltransferase activity in myeloproliferative disease [38]. In contrast, phosphorylation of MEP50 by Cyclin D1/CDK4 kinase enhanced PRMT5/MEP50 activity of histone methylation in the nucleus [39]. Moreover, Lattouf H. et al. found that LKB1-mediated phosphorylation of PRMT5 promotes its interaction with MEP50, pICln, and RioK1, leading to PRMT5 activation in breast cancer cells [40]. Additionally, several other kinases are also involved in PRMT5 regulation, including AKT, serum- and glucocorticoid-inducible kinases (SGK), and protein kinase C ι (PKCι) [41,42]. Aside from phosphorylation, a study showed that the E3 ubiquitin ligase CHIP negatively regulates PRMT5 expression by promoting PRMT5 degradation through K48-linked ubiquitination in the presence of the HSP90 inhibitor 17-AAG in prostate cancer cells [43]. 

In the current study, we identified that TRAF6 acts as an E3 ubiquitin ligase to catalyze non-proteolytic K63-linked ubiquitination of PRMT5 and subsequently promote PRMT5 activation. Our findings elucidate a novel ubiquitination-dependent regulatory mechanism of PRMT5. We are aware that additional evidence, such as in vitro ubiquitination data, is required to further support TRAF6 as a direct E3 ubiquitin ligase of PRMT5. Moreover, as a versatile protein, PRMT5 methylates numbers of substrates to regulate many fundamental cellular processes, including DNA repair, transcription, and cell signaling transduction. Further studies are warranted to investigate the impacts of TRAF6-mediated ubiquitination in regulating these cellular processes downstream of PRMT5. For example, it will be critical to explore how PRMT5 ubiquitination affects H4R3me2s-mediated transcription. Furthermore, growing evidence indicates that a protein can be modified by various PTMs simultaneously to orchestrate the protein function [44]. It would be interesting to determine the crosstalk among different PTMs of PRMT5, such as ubiquitination and phosphorylation, which will provide another higher layer of regulation.

TRAF6 belongs to the tumor necrosis factor receptor-associated factor (TRAF) family and functions as either an adaptor protein or E3 ligase to regulate cell signaling transduction, such as the NF-κB pathway [27], AKT signaling [45], and TGF-β signaling [46,47,48]. In this study, we further extend its role in PRMT5 regulation, revealing crosstalk between arginine methylation and ubiquitination. Interestingly, PRMT5 also directly regulates the activation of the NF-κB pathway, AKT signaling, and TGF-β signaling by catalyzing the symmetric demethylation on the p65 subunit of NF-κB, AKT, and SMAD4, respectively [17,18,49]. It will be worth investigating how TRAF6 and PRMT5 coordinatively control these signaling pathways to regulate various biological processes, cell proliferation, migration, and tumor growth.

Both PRMT5 and TRAF6 are frequently overexpressed in various types of human cancers, including breast cancer, colorectal cancer, gastric cancer [50]. Their high expression correlated with poor prognosis, survival, and chemotherapeutic resistance in cancer patients [50,51,52,53,54]. Therefore, both PRMT5 and TRAF6 are considered as attractive therapeutic targets. Multiple PRMT5 inhibitors have been developed, such as GSK3326595 [55], JNJ-64619178 [56], and MRTX1719 [57], all of which significantly suppressed tumor growth in preclinical models and are being currently tested in clinical trials [58]. The development of TRAF6 inhibitors is still in the early stage. For example, the compound C25–140 directly binds to TRAF6 to prevent TRAF6 interaction with the E2 Ubc13, thereby inhibiting the formation of K63-linked ubiquitin chain on targets [33]. However, the potency of C25–140 is low and its role in tumor growth has yet to be determined. Our study provides preliminary evidence to show that a combination of PRMT5 and TRAF6 inhibitors achieves a better anti-proliferative effect in breast cancer cells. Further studies in breast cancer mouse models are warranted to validate this notion.

## 5. Conclusions

TRAF6 interacts with and catalyzes K63-linked ubiquitination of PRMT5, which in turn promotes PRMT5-MEP50 complex formation, activation, cell proliferation, and tumor growth (Figure 6L). These results reveal a possible molecular mechanism underlying how PRMT5 activity and function is regulated by a non-proteolytic ubiquitination signal.

## Data Availability

All data needed to evaluate the conclusions of this study are present in the paper.

## Supplementary Materials

The following supporting information can be downloaded at: https://www.mdpi.com/article/10.3390/cancers15092501/s1, Original blots images for Figure 1, Figure 2, Figure 3, Figure 4, Figure 5 and Figure 6.
