# Supplementary material for: TRAF6 Promotes PRMT5 Activity in a Ubiquitination-Dependent Manner
Source: Cancers (Basel). 2023 Apr 27;15(9):2501. doi: 10.3390/cancers15092501 (PMC10177089; doi:10.3390/cancers15092501)

Original blots images for Figure 1

Fig. 1A

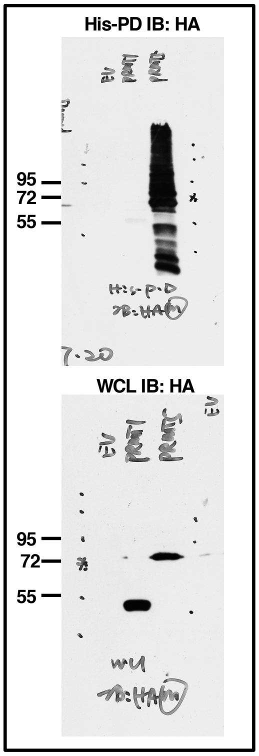

Fig. 1B

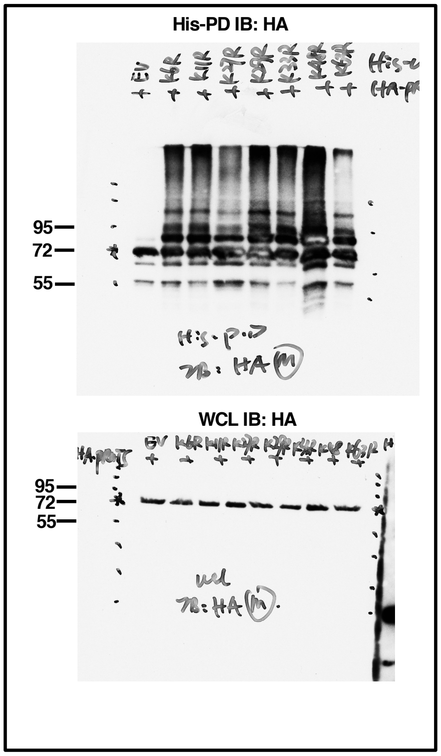

Fig. 1C

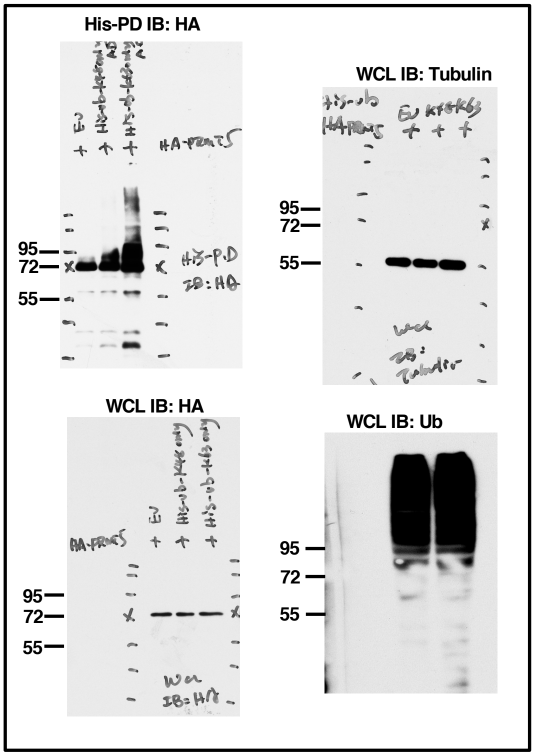

Fig. 1D

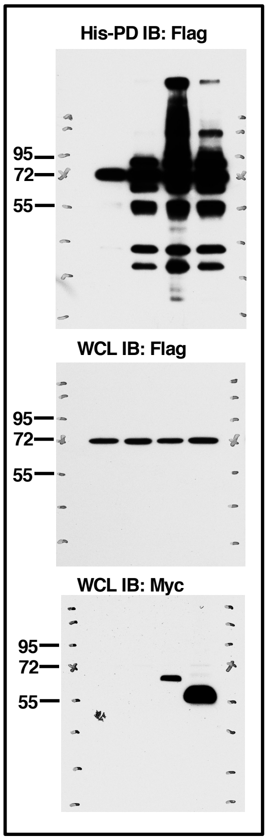

Fig. 1E

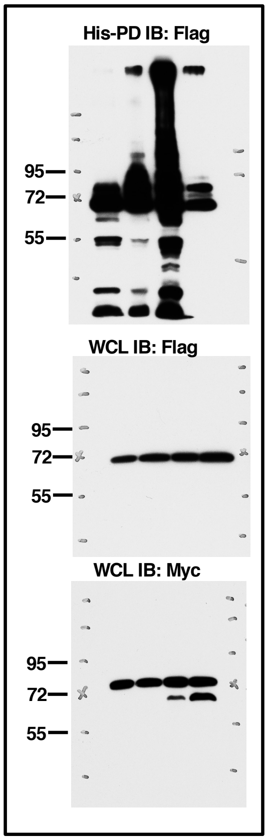

Fig. 1F

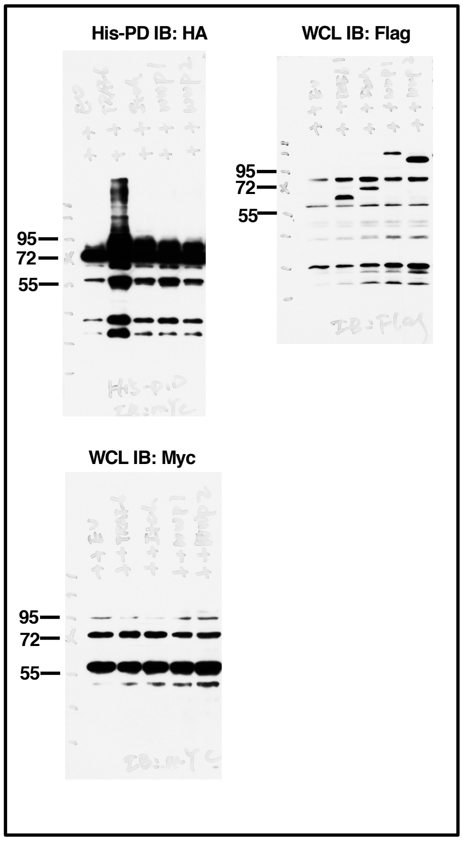

Original blots images for Figure 2

Fig. 2A

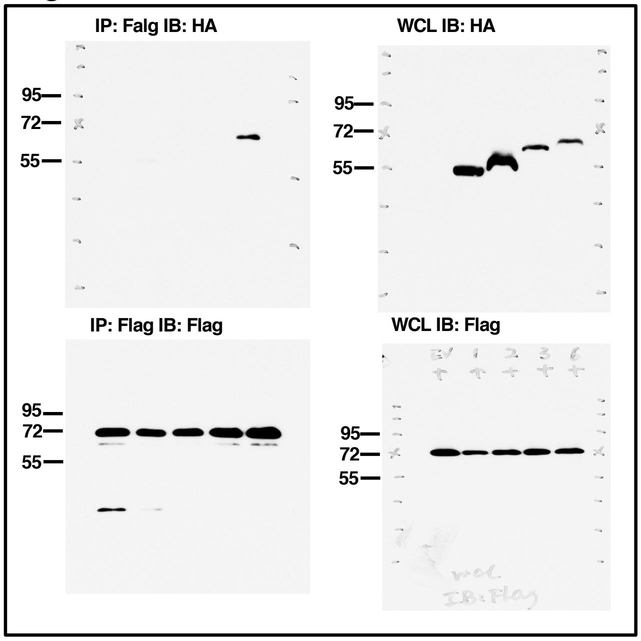

Fig. 2B

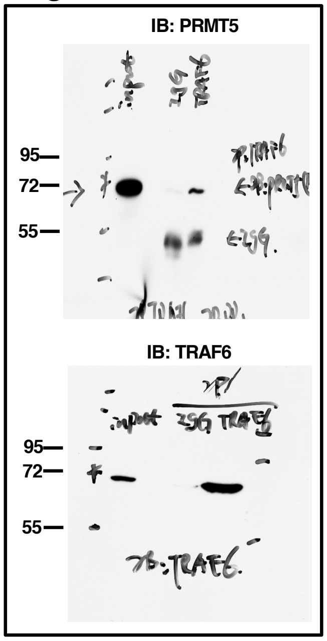

Fig. 2C

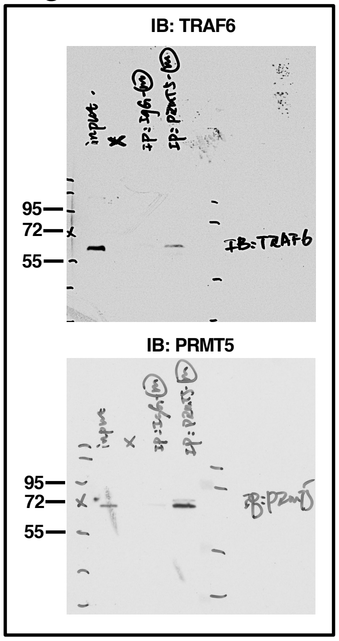

Fig. 2E

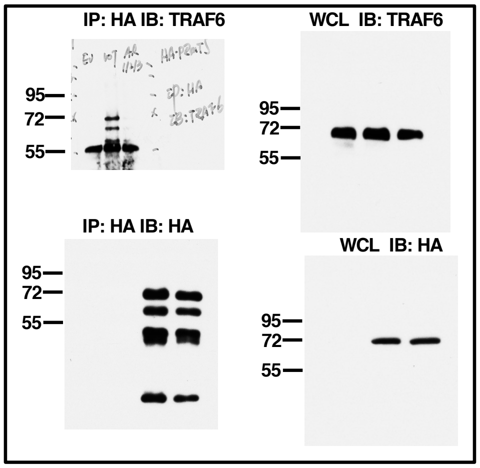

Fig. 2F

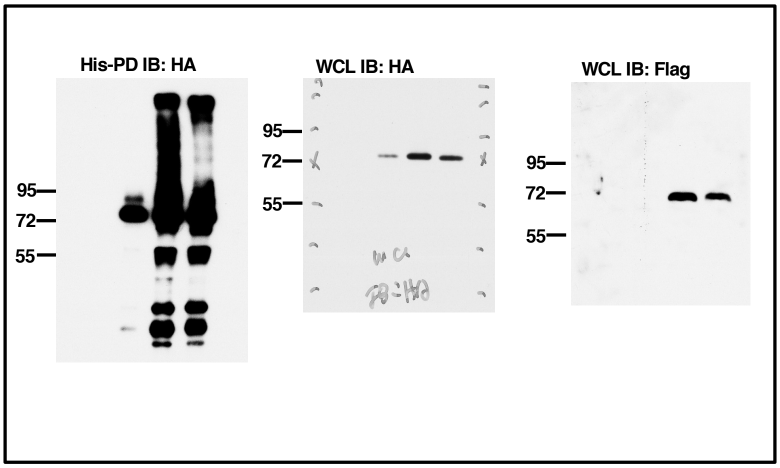

Original blots images for Figure 3

Fig. 3B

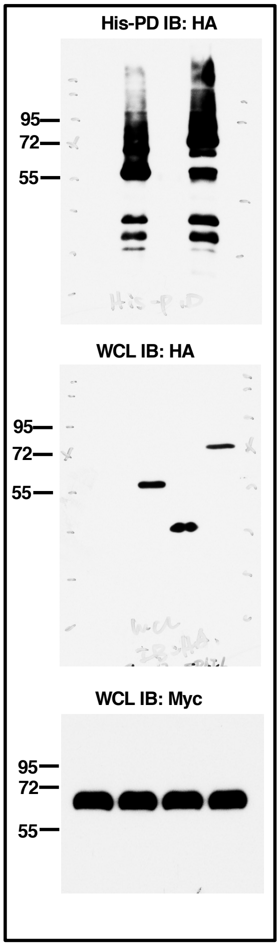

Fig. 3C

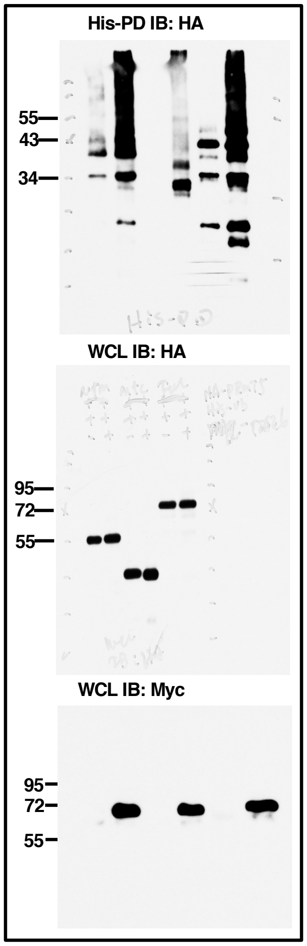

Fig. 3E

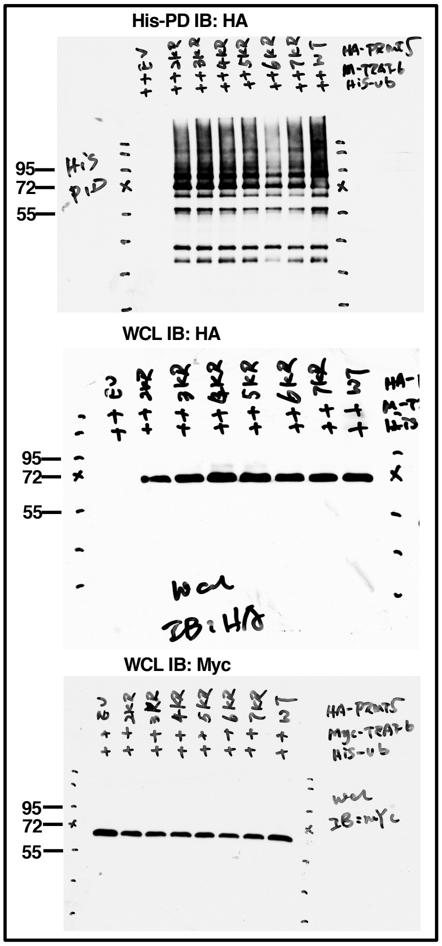

Fig. 3F

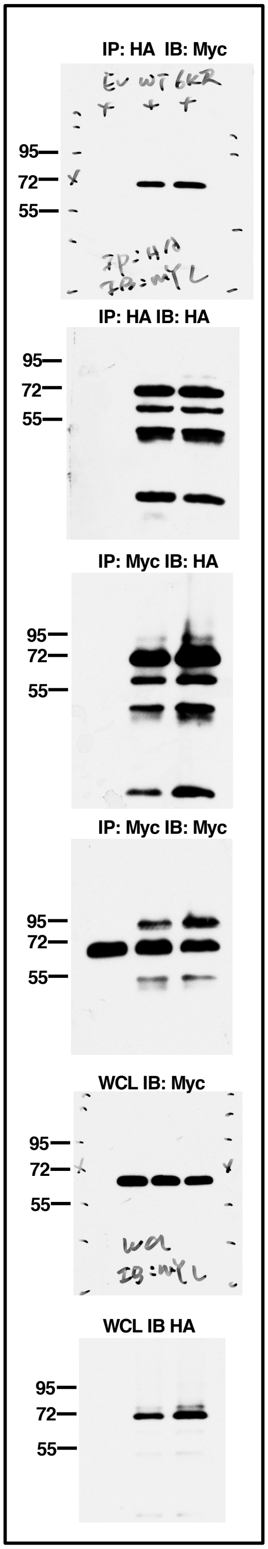

Original blots images for Figure 4

Fig. 4A

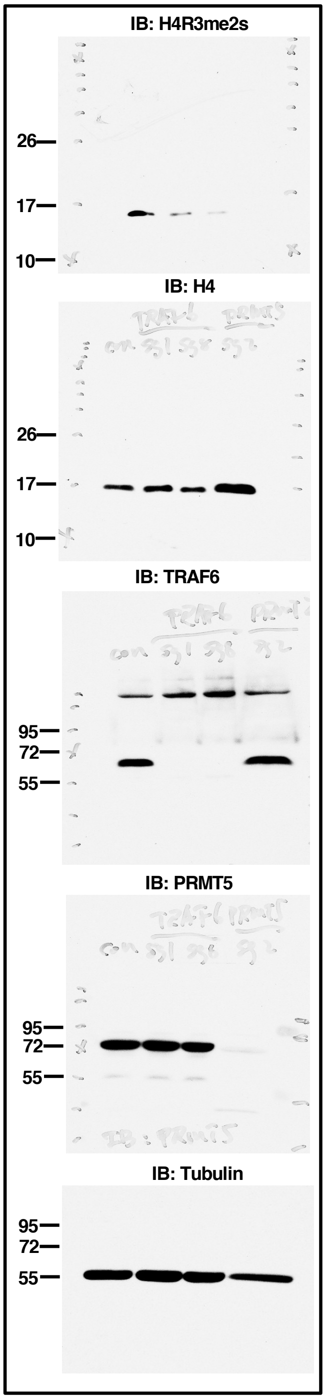

Fig. 4B

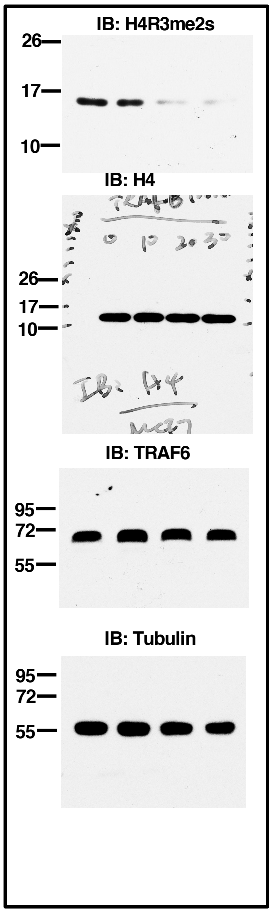

Fig. 4C

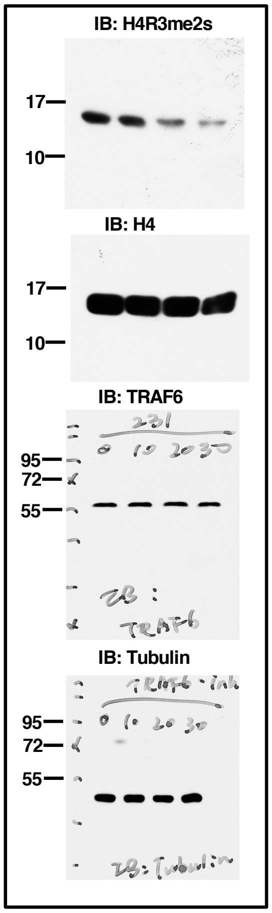

Fig. 4D

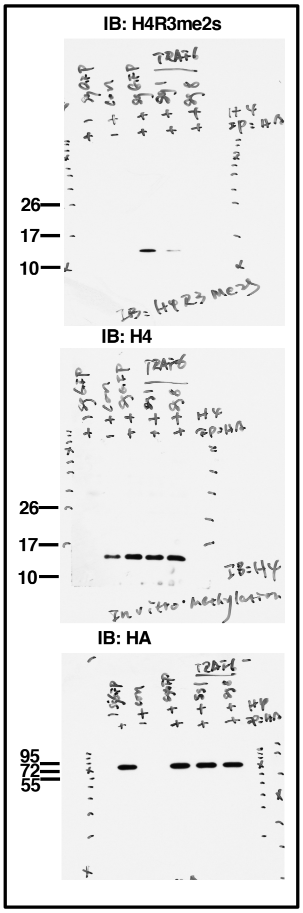

Fig. 4E

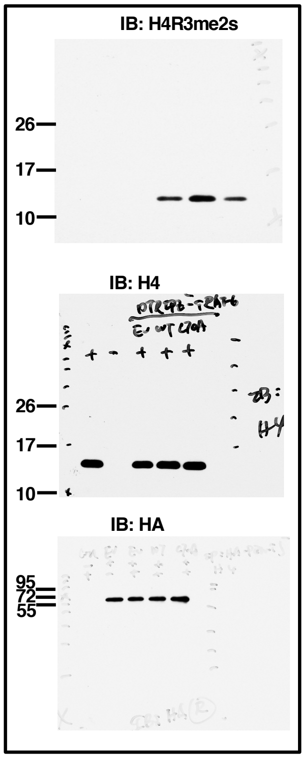

Fig. 4I

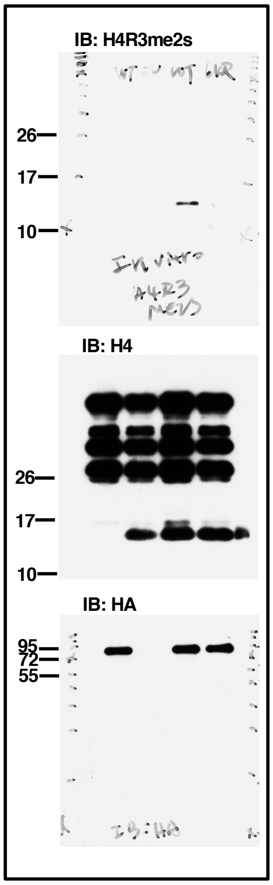

Fig. 4F

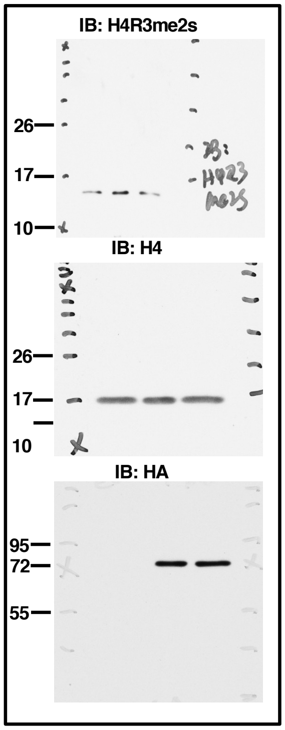

Fig. 4G

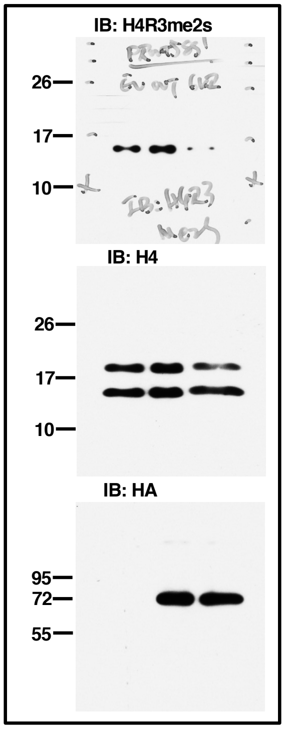

Fig. 4H

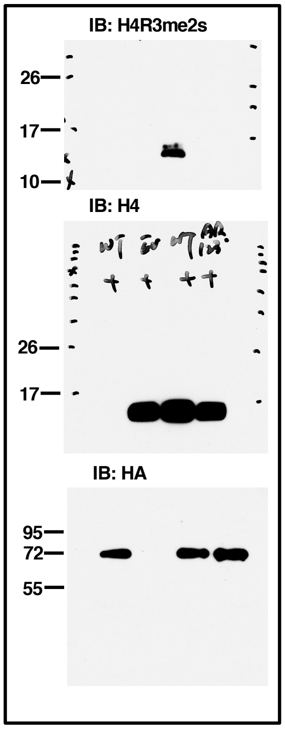

Original blots images for Figure 5

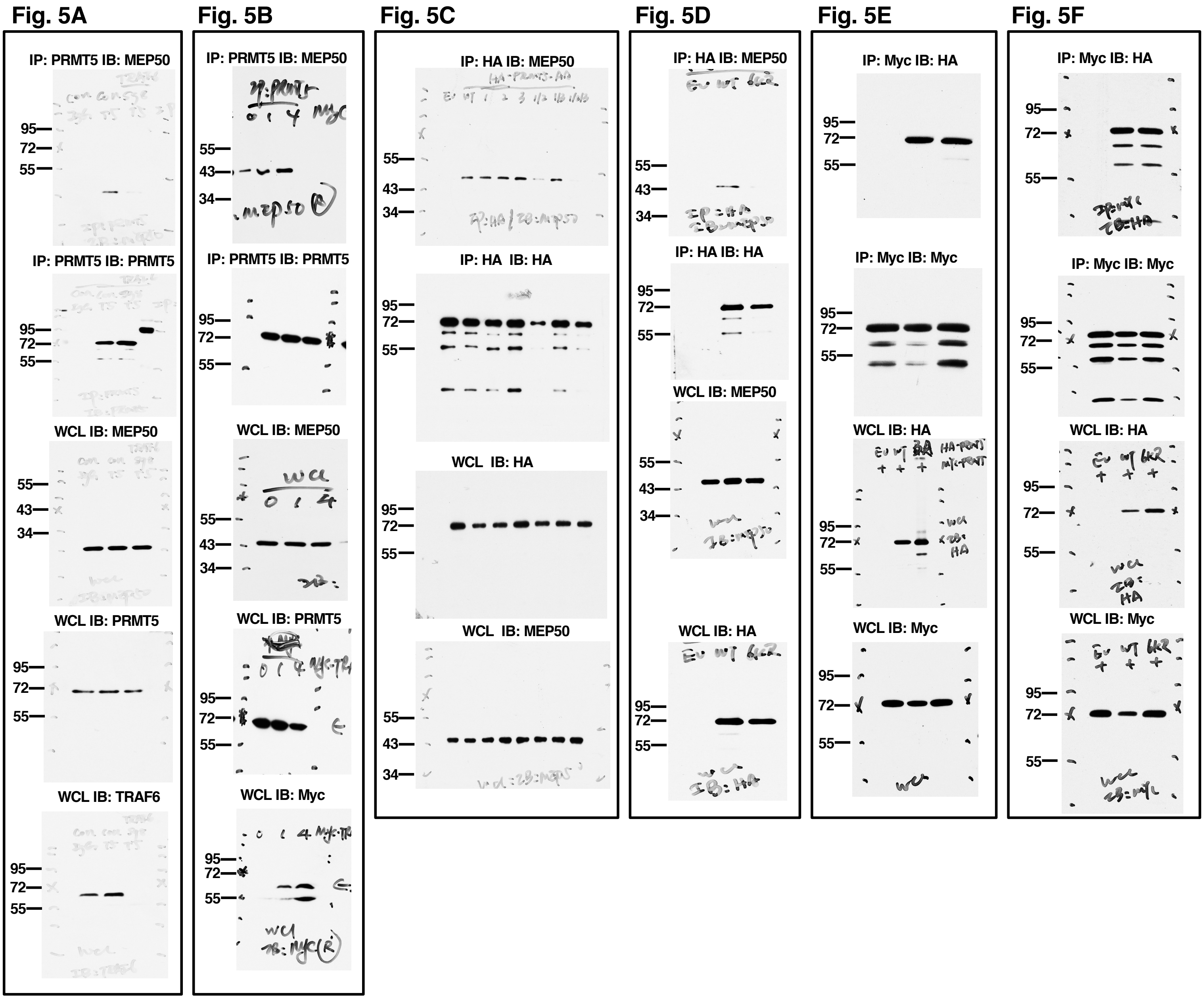

Original blots images for Figure 56

Fig. 6K

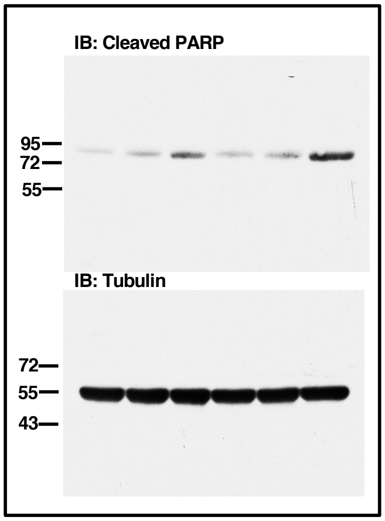

Supplement: Supplementary file 1 [file cancers-15-02501-s001.zip › cancers-2311466-supplementary.pdf]
